# Biologically Inspired Methods for Imaging, Cognition, Vision, and Intelligence

**DOI:** 10.1155/2016/2402067

**Published:** 2016-05-18

**Authors:** Yufeng Zheng, Erik Blasch, Adel S. Elmaghraby

**Affiliations:** ^1^Department of Advanced Technologies, Alcorn State University, Lorman, MS 39096, USA; ^2^US Air Force Research Laboratory (AFRL), Rome, NY 13441, USA; ^3^Department of Computer Engineering and Computer Science, University of Louisville, Louisville, KY 40292, USA

State-of-the-art image analysis and pattern recognition techniques have been successfully applied to a wide variety of industrial fields such as medical imaging, remote sensing, and biometrics, as well as methods for machine learning, computer vision, and visualization. However, the current methods of image analysis, computer vision, and artificial intelligence cannot achieve the performance of human visual and cognitive abilities although advances have progressed in hardware including optics, electronics, and computers. As Scott [1] stated, “the evolution of biological vision is gradual (incremental) and top down with basic modules and primitive architectural structures preserved.” The future of computer vision will be biologically inspired [2] that has much in common with human vision as a unified science of vision [1]. Thus, computational photography, cognition, and intelligence have recently become the sources of interactive scientific research.

This special issue focuses on biologically inspired or nature-driven approaches to computer vision. There were a total of 17 manuscripts received, five of which were accepted for publication. Two papers address computer vision problems: estimation of homographs between two different perspectives and computation of visual saliency. The other three papers present optimization algorithms for pattern recognition using an artificial bee colony approach, a differential evolution method, and a feed-forward neural network.

The success of computer vision and intelligence may start from the data representation of the real world (signal sensing, imaging), through analysis and cognition (learning, modelling, and classification), to achieve a certain level of performance (model revision, autolearning, and improvement). This special issue sheds light on the importance of bioinspired methods. Furthermore, editors believe that deeper research and development on bioinspired methods will greatly benefit the computational intelligence community.

*Yufeng Zheng* *Erik Blasch* *Adel S. Elmaghraby*
